# Defining the incremental value of 3D T2-weighted imaging in the assessment of prostate cancer extracapsular extension

**DOI:** 10.1007/s00330-019-06070-6

**Published:** 2019-03-18

**Authors:** Iztok Caglic, Petra Povalej Brzan, Anne Y. Warren, Ola Bratt, Nimish Shah, Tristan Barrett

**Affiliations:** 1 Department of Radiology, Norfolk & Norwich University Hospital, Colney Lane, Norwich, Norfolk NR4 7UY UK; 2 Department of Radiology, Addenbrooke’s Hospital and University of Cambridge, Cambridge, UK; 3 University of Ljubljana, Faculty of Medicine, Ljubljana, Slovenia; 4 University of Maribor, Faculty of Health Sciences, Maribor, Slovenia; 5 University of Maribor, Faculty of Electrical Engineering and Computer Science, Maribor, Slovenia; 6 Department of Histopathology, Addenbrooke’s Hospital and University of Cambridge, Cambridge, UK; 7 Department of Urology, Addenbrooke’s Hospital and University of Cambridge, Cambridge, UK; 8 CamPARI Clinic, Addenbrooke’s Hospital and University of Cambridge, Cambridge, UK

**Keywords:** Magnetic resonance imaging, 3D imaging, Prostate cancer, Tumour staging

## Abstract

**Objectives:**

To assess the added value of 3D T2-weighted imaging (T2WI) over conventional 2D T2WI in diagnosing extracapsular extension (ECE).

**Methods:**

Seventy-five patients undergoing 3-T MRI before radical prostatectomy were included. PI-RADS ≥ 4 lesions were assessed for ECE on 2D T2W images using a 5-point Likert scale (1 = no ECE, 5 = definite ECE) and the length of tumour prostatic capsular contact. A second read using 3D T2W images and reformats evaluated ECE and the maximal 3D capsular contact length and surface.

**Results:**

One hundred six lesions were identified at MRI. ECE was confirmed by histology in 54% (57/106) of lesions and 64% (48/75) of patients. Sensitivity and specificity for 3D T2 reads were 75.4% versus 64.9% (*p* = 0.058), respectively, and 83.7% versus 85.7% (*p* = 0.705) for 2D T2 reads, respectively. 3D T2W reads showed significantly higher mean subjective Likert scores of 3.7 ± 1.4 versus 3.3 ± 1.4 (*p* = 0.001) in ECE-positive lesions and lower mean Likert score of 1.5 ± 1 versus 1.6 ± 0.9 (*p* = 0.27) in ECE-negative lesions compared with 2D T2W reads. 3D contact significantly increased sensitivity from 59.6 to 73.7% (*p* = 0.03), whilst maintaining the same specificity of 87.8% (*p* = 1). High-grade group tumours (≥ Gleason 4 + 3) showed significantly higher ECE prevalence than low-grade tumours (88% versus 44%, *p* < 0.001) and a positive predictive value (PPV) for ECE of 90.9% with ≥ 5 mm of contact versus PPV of 90.4% at ≥ 12.5 mm for lower grade tumours.

**Conclusions:**

3D T2WI significantly increases sensitivity and confidence in calling ECE. The capsular contact length threshold differed between low- and high-grade cancers.

**Key Points:**

*• 3D capsular contact length and 3D surface contact significantly increased sensitivity in diagnosing ECE.*

*• 3D T2W reads significantly increased reader confidence in calling ECE.*

*• Thresholds for capsular contact length differed between low-grade and high-grade cancers.*

**Electronic supplementary material:**

The online version of this article (10.1007/s00330-019-06070-6) contains supplementary material, which is available to authorized users.

## Introduction

Prostate cancer (PCa) is the commonest malignancy in men and the second leading cause of male cancer-related mortality [1]. Accurate local staging is essential for treatment planning and prognosis in patients where the aim is for curative treatment [2]. The principal aim is to differentiate between organ-confined disease and extracapsular extension (ECE) which is associated with higher rate of positive surgical margins, biochemical recurrence and micro-metastatic disease [3, 4]. The presence of ECE by itself does not preclude radical treatment; however, preoperative identification is important to allow for wider surgical margins on the affected side [5], or for more aggressive management to be delivered to patients undergoing external beam radiotherapy [6].

Magnetic resonance imaging (MRI) has been shown to have higher accuracy for prediction of ECE than clinical nomograms, and staging with digital rectal examination (DRE), PSA and biopsy Gleason score [7–10]. However, the true sensitivity and specificity are unknown as gross ECE on MRI often precludes surgery, denying the opportunity for gold standard histopathological confirmation. A recent meta-analysis has shown only moderate overall performance using a standard subjective MRI interpretation, mainly due to a limited sensitivity [11]. Several studies have therefore evaluated different approaches to improve sensitivity, such as use of endorectal coils (ERC) [12, 13], risk-probability scoring using ESUR criteria [14], adding functional MRI sequences [15], or measuring tumour contact with the capsule [16–18]. Capsular contact length appears to be a promising predictive marker, but the reported threshold value varies significantly between studies from 6 to 20 mm [16–18], whilst PI-RADS 2.0 guideline suggest a cutoff of 10 mm for assessing ECE [19].

3D fast spin echo (FSE) with variable flip angle (“CUBE”) MR imaging allows for reconstruction in multiple planes using voxels acquired isotropically with no gap. The partial volume effect is thereby minimised, resulting in improved spatial resolution and potentially better diagnostic performance as previously shown in the liver, uterus and knee [20–22]. Measurement in 3D planes may thus allow for more accurate delineation of prostate capsular contact, and reformatting may help assess for ECE, analogous to the axial oblique imaging acquired for rectal and cervical cancer MRI staging [23, 24]. Rosenkrantz et al [25] and Itatani et al [26] have both compared the diagnostic performance of isotropic 3D T2 sequences. Whilst the first group found no significant difference between the 2D and 3D sequences in diagnosing T3a disease at 3 T, the latter reported improved sensitivity of 3D T2 at 1.5-T imaging [25, 26]. In addition, good performance of 3D imaging in staging of prostate cancer has been described by Cornud et al [27] and Matsuoka et al [28]; however, neither study directly compared the results to conventional 2D T2WI.

The aims of this study were therefore to provide further evidence on the performance of 3-T MRI for local prostate cancer staging and to assess the added value of 3D T2W imaging.

## Methods

This single-institution retrospective study was approved by the local ethics committee (HBREC.2016.08) with the need for obtaining informed consent waived. Seventy-five consecutive patients who underwent preoperative MRI and subsequent radical prostatectomy for prostate cancer between September 2014 and January 2017 were included. Inclusion criteria included consecutive patients undergoing 3-T MRI at our institution prior to prostatectomy. Pre-determined exclusion criteria were applied for total hip replacement (*n* = 2) and any previous treatment for prostate cancer (*n* = 0).

### Magnetic resonance imaging

All patients underwent 3-T MRI (MR750, GE Healthcare) using a 32-channel phased-array coil. Sequences included high-resolution T2-weighted 2D fast recovery FSE images of the prostate in axial, sagittal and coronal planes and sagittal T2 3D FSE sequences (Table 1). Additional sequences included axial T1-weighted imaging: TR/TE 561/11 ms, FOV 24 × 24 cm, resolution 1.1 × 1.0 mm; axial diffusion-weighted imaging (DWI) dual spin echo-planar imaging: TR/TE 3775/70 ms, FOV 28 × 28 cm, resolution 2.2 × 2.2 mm, with 6 signal averages; *b*-values of 150, 750, 1400 and 2000s/mm^2^; and axial 3D dynamic contrast–enhanced (DCE) imaging: fast-spoiled gradient echo sequence (TR/TE 4.088/1.788 ms; FOV 24 × 24 cm) following a bolus of Gadobutrol (Gadovist, Schering AG); dose 0.1 mmol/kg. Axial T2, DWI and DCE sequences were spatially matched, with a 3-mm thickness and 0-mm gap.

**Table 1:** T2 Sequences in MRI protocol

Parameter	Axial 2D T2 FSE	Sagittal 3D T2 FSE
TR/TE (ms)	3712/102	3000/117
Flip angle (°)	111	N/A
ETL	16	90
Averages	3	2
Section thickness (mm)	3	1
Section gap (mm)	0	0
FOV (mm)	220 × 220	220
Resolution (mm)	0.85 × 0.57	1.0 × 0.8
Acquisition time (min:s)	04:39	3:13

*FSE* fast spin echo, *FOV* field of view, *ETL* echo train length

### Image analysis

Images were reviewed by a uro-radiologist (T.B.), blind to the clinical details, with 8 years’ experience in prostate MRI reporting.

#### First read

PI-RADS version 2 was used to score suspicious MRI lesions [29]. Lesions with a score of ≥ 4 were subjectively assessed for the presence of ECE on 2D T2WI using a 5-point Likert scale: 1 = no ECE, 2 = ECE unlikely, 3 = possible ECE, 4 = probable ECE, 5 = definite ECE. This was based on the following criteria: (a) capsular abutment; (b) capsular irregularity, retraction, or thickening; (c) neurovascular bundle thickening; (d) capsular signal loss or bulging and (e) measurable extracapsular disease. For the purposes of statistical analysis, scores of ≥ 3 were considered as ECE. In addition, the length of contact between lesion and prostatic capsule was measured in axial, sagittal and coronal planes, with the “maximum 2D contact” representing the longest of these.

#### Second read

The presence of ECE was evaluated on 3D T2 and its reformats at least 4 weeks after the first reads. The same Likert scale was used for subjective assessment. As 3D allows indefinite reformat planes, a maximum length of contact between the lesion and the prostatic capsule was measured (3D contact). An approximation of the surface contact (3D surface) was then calculated using the formula for area of an ellipse (*π* × *a* ∕ *2* × *b* ∕ *2)* where *a* stands for maximal 3D contact and *b* for contact perpendicular to it. Both 3D contact and 3D surface are novel quantitative parameters to predict extracapsular extension and to our knowledge have not been described before (Supplemental Figure 1).

### Pathological analysis

Whole-mount radical prostatectomy specimens were used as the reference standard for extracapsular extension. Tumour was outlined on haematoxylin and eosin (H&E)–stained sections from each slice by an experienced genitourinary pathologist specialising in prostate cancer (A.Y.W.). Prostatectomy specimens were fixed in formalin and oriented by the location of the seminal vesicles, posterior surface of the prostate and the position of the urethra. ECE was considered to represent any presence of the cancer cells in the periprostatic soft tissue; this was subcategorised as “focal” when it extended < 0.5 mm beyond the prostatic capsule and “established” when ≥ 0.5 mm [30].

## Statistical analysis

Reader’s assessments of ECE were compared with the pathologic reference on a lesion-per-lesion basis. Standard performance statistics of sensitivity, specificity, positive predictive value (PPV) and negative predictive value (NPV) were calculated for ECE, based on a Likert score ≥ 3 being a positive result. McNemar’s test was used to compare sensitivities and specificities and Kosinski test to compare PPVs and NPVs. Receiver operating characteristic (ROC) analysis was then used to assess the diagnostic utility associated with the measured length of capsular contact from 2D and 3D for the detection of ECE. In particular, the ROC analysis was performed to identify the optimal threshold for the length and surface of different capsular contact types. The Youden index was used to define a single optimal threshold maximising the average of sensitivity and specificity. The DeLong test was used to compare two AUC curves. Likert scores for ECE-positive and ECE-negative lesions between the 2D and 3D reads were compared with exact the Wilcoxon-Pratt signed-rank test. For the purposes of analysing contact thresholds separately, we considered a low-grade group (LGG) where the Gleason score was ≤ 3 + 4 (grade groups 1–2) and a high-grade group (HGG) with Gleason score ≥ 4 + 3 (grade groups 3–5) [31]. Prevalence of ECE between the two groups was assessed with Fischer’s exact test. Intraclass correlation coefficient (ICC) was computed using a two-way mixed effects model to separately test reliability of 3D contact and 3D surface measurements. ICC values were defined as indicating poor (< 0.5), moderate (0.5–0.75), good (0.75–0.90) and excellent reliability (> 0.90) [32]. In addition, simple kappa (*k*) coefficients were used to separately assess the agreement between the 3D contact and 3D surface measurements at the derived optimal ROC thresholds, where 0–0.20 = slight, 0.21–0.40 fair, 0.41–0.60 moderate, 0.61–0.80 substantial and 0.81–1 almost perfect agreement [33].

A value of *p* < 0.05 was considered statistically significant. Statistical analyses were performed using IBM SPSS software, version 24.0.

## Results

Median patient age was 64.5 years (IQR 57.2–67.0) and median PSA was 8.5 (IQR 5.7–10.4). Interval between MRI and subsequent biopsy was 22 days (IQR 15–50, range 7–71). Median time between MRI and radical prostatectomy was 81 days (IQR 54–114). A total of 106 lesions with PI-RADS score of ≥ 4 were identified and analysed for the presence of extracapsular extension. ECE was confirmed by histology in 54% (57/106) of lesions with PI-RADS score of ≥ 4 and in 64% (48/75) of patients out of which 19% (7/48) also harboured T3b disease (Table 2). ECE was reported as focal (< 0.5 mm) in 28% (16/57) and established in 72% (41/57) of lesions with ECE. The MRI-designated index lesions had the highest stage in all cases; second or third lesions were non-additive to staging on a patient-level basis as none of these lesions upgraded the final T stage.

**Table 2:** Demographic data and histopathological findings at radical prostatectomy

Characteristic	Summary
Age (median, years)	64.5 (57.2–67.0)
PSA (median, ng/ml)	8.5 (5.7–10.4)
Highest biopsy Gleason score	No. Pts. (%)
6	6 (8%)
7 (3 + 4)	44 (59%)
7 (4 + 3)	6 (8%)
8	10 (13%)
9 (4 + 5)	8 (11%)
10	1 (1%)
Final Gleason score	No. Pts. (%)
6	6 (8%)
7 (3 + 4)	40 (53%)
7 (4 + 3)	12 (16%)
8	8 (11%)
9 (4 + 5)	9 (12%)
Final T stage	No. Pts. (%)
T2	27 (36%)
T3a	41 (55%)
T3b	7 (9%)

### Subjective reads

**Fig. 1:**
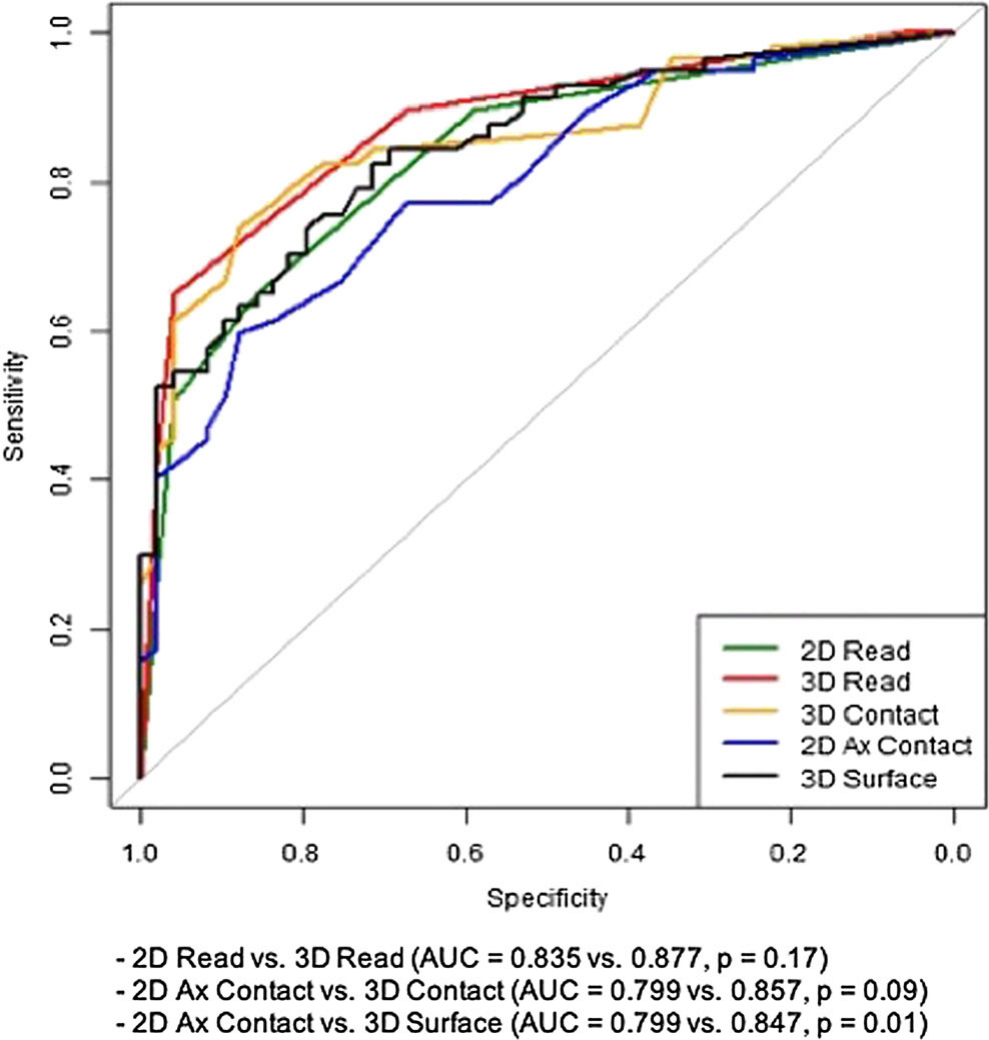
ROC curves based on the reads using conventional FSE T2W (2D), 3D CUBE T2W (3D) imaging and different capsular contacts. Tumour contact length determined on axial 2D T2WI (2D Ax Contact). Maximal tumour contact length determined on 3D T2WI (3D Contact). Tumour surface contact derived from two measurements on 3D T2WI (3D Surface). Reference line indicates AUC = 0.5

Receiver operating characteristic (ROC) curve analysis for the prediction of ECE revealed an area under the ROC curve (AUC) of 0.877 (95% CI, 0.813–0.941) for 3D T2W imaging and 0.835 (95% CI, 0.761–0.909) for conventional 2D T2W imaging (*p* = 0.17) (Fig. 1**)**. Sensitivity and specificity for 3D T2 were 75.4% versus 64.9% (*p* = 0.058), respectively, and 83.7% versus 85.7% (*p* = 0.705) for 2D T2, respectively. Positive predictive value (PPV) and negative predictive value (NPV) were 84.3% versus 84.1% (*p* = 0.963) for 3D, and 74.6% versus 67.7% (*p* = 0.094) for 2D, respectively.

**Fig. 2:**
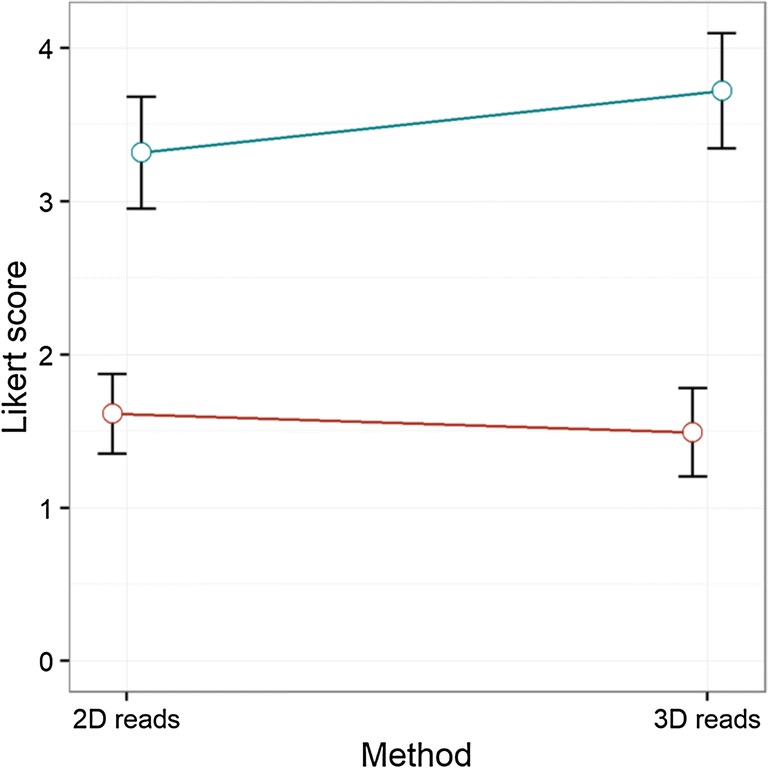
Mean Likert score (95% CI) between 2D and 3D reads for pathological (blue) and non-pathological (red) ECE

**Fig. 3:**
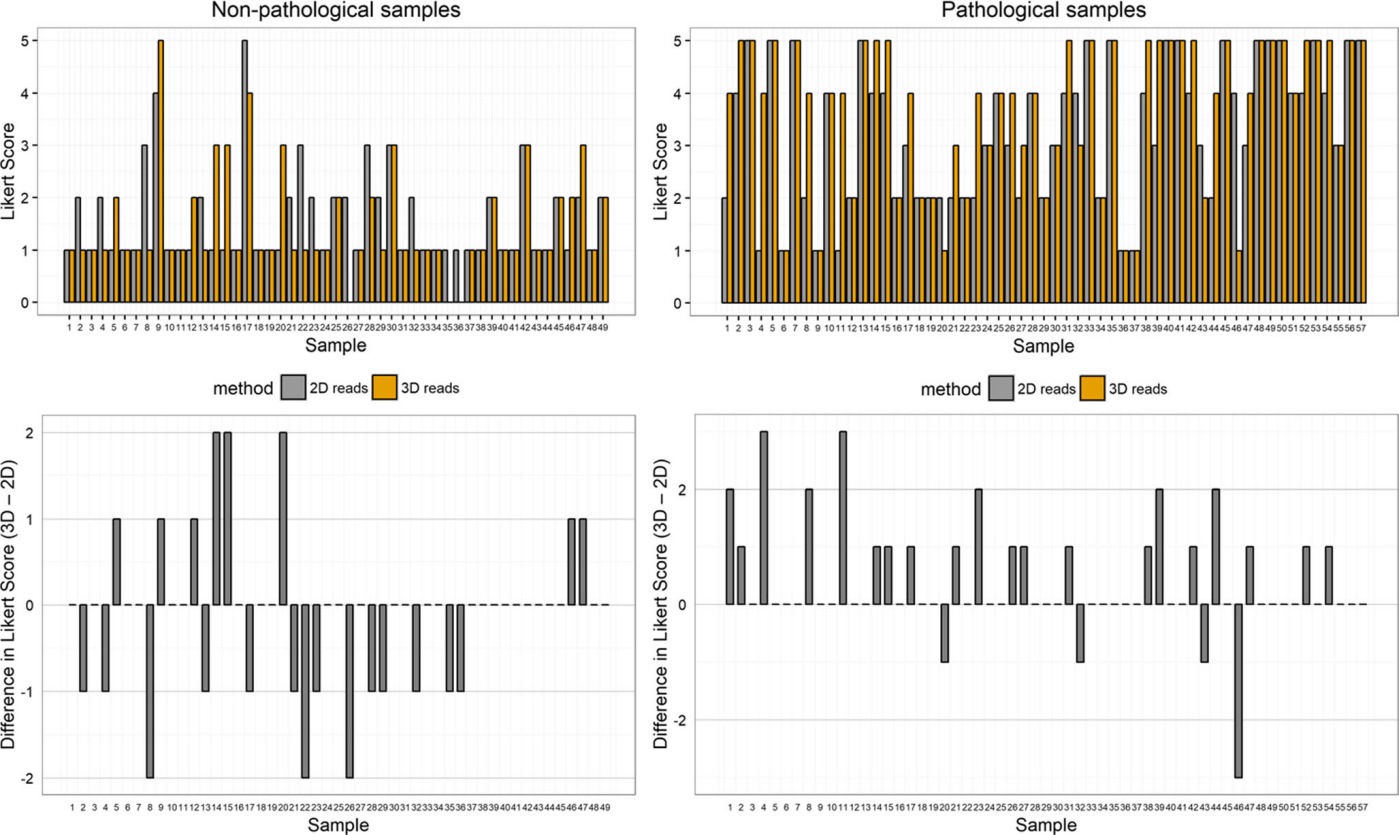
Non-pathological samples: Likert score for lesions with no ECE at 2D and 3D reads and their difference. Pathological samples: Likert score for lesions with ECE at 2D and 3D reads and their difference

In addition, 3D T2 reads showed a significantly higher mean Likert score of 3.7 ± 1.4 versus 3.3 ± 1.4 (*p* = 0.001) in ECE-positive lesions and non-significantly lower Likert score for ECE-negative lesions with average score of 1.5 ± 1 versus 1.6 ± 0.9 (*p* = 0.272) as compared with 2D T2 (Figs. 2 and 3).

### Different contact types

For detection of ECE, 3D surface was significantly better than conventionally measured capsular contact at axial 2D imaging with AUC of 0.847 (95% CI, 0.775–0.920) versus 0.799 (95% CI, 0.716–0.882) (*p* = 0.01). 3D surface significantly had increased sensitivity from 59.6 to 82.5% (*p* < 0.001) but this was associated with a loss of specificity from 87.8 to 71.4% (*p* < 0.001). 3D contact with AUC of 0.857 (95% CI, 0.785–0.929) was also superior to 2D contact (0.799) but this did not reach statistical significance (*p* = 0.092) (Fig. 1). 3D contact had significantly higher sensitivity than 2D contact at 73.7% versus 59.6% (*p* = 0.03), whilst maintaining the same specificity of 87.8% (*p* = 1). There was a significant difference between the mean 3D contact (10.7 mm) and 2D contact (12.1 mm) (*p* = 0.02). Youden index–derived thresholds for 2D contact, 3D contact and 3D surface were 13.5 mm, 10.5 mm and 40.0 mm^2^, respectively. The shortest derived threshold was in vertical plane (either coronal or sagittal) at 5.5 mm. Table 3 shows the AUC, sensitivity, specificity, PPV and NPV and thresholds for different capsular contacts.

**Table 3:** Performance of different contacts at optimal thresholds

Contact type	AUC	Sensitivity	Specificity	PPV	NPV	Cutoff^a^
Axial contact (2D)	0.799	0.596	0.878	0.850	0.651	13.5 mm
Coronal or sagittal contact (2D)	0.801	0.754*	0.776	0.796	0.731	5.5 mm
Max contact (2D)	0.808	0.614	0.878	0.854	0.662	13.5 mm
3D contact	0.857	0.737*	0.878	0.875	0.741*	10.5 mm
3D surface	0.847*	0.825*	0.714*	0.770	0.778*	40.0 mm^2^

*Significant difference in comparison to axial contact (2D) (*p* < 0.05)

^a^Youden index–derived threshold

### Reliability and agreement of 3D measurements

ICC for 3D contact was 0.97 (95% CI, 0.96–0.98) and for 3D surface 0.99 (95% CI, 0.98–0.99), indicating excellent reliability for both parameters. In addition, substantial agreement was achieved for 3D contact (*k* = 0.79; 95% CI 0.67–0.91) and almost perfect for 3D surface (*k* = 0.92; 95% CI, 0.85–1).

### Low- versus high-grade subgroups

A significant difference was observed between performance of 2D contact and 3D contact in LGG with AUC 0.840 (95% CI, 0.749–0.931) for 3D and 0.741 (95% CI, 0.632–0.849) for 2D reads (*p* = 0.03), whereas in HGG, there was no statistical difference with AUC at 0.904 (95% CI, 0.791–1.00) versus 0.909 (95% CI, 0.798–1.00), respectively. Additional analysis of 3D reads revealed that HGG tumours showed significantly longer mean capsular contact when compared with LGG tumours at 15.3 ± 9.8 mm versus 9.0 ± 8 mm, respectively, (*p* = 0.001), and significantly higher ECE prevalence, which was present in 23/28 (88%) cases of HGG compared with 34/78 (44%) of cases of LGG (*p* < 0.001). After applying the PI-RADS 2.0 guideline suggested threshold of 10 mm for assessing ECE, biopsy-confirmed LGG index lesions at 10 mm or below showed established ECE in 3% (1/35) whereas in HGG this was present in 41.6% (5/12) index lesions (*p* = 0.01). Based on surgical pathology results, LGG lesions at 10 mm or below showed ECE in 21% (10/48) whereas in HGG this was present in 50% (5/10) lesions (*p* = 0.106). In addition, in HGG, 100% specificity and PPV were achieved for capsular contact of > 8 mm and a PPV of 90.9% at ≥ 5 mm; conversely for LGG, a PPV of 90.4% was achieved at a cutoff at ≥ 12.5 mm and a contact at 20.0 mm was needed to reach a specificity and PPV of 97.7% and 88.8%, respectively (Supplemental Tables 1 and 2).

## Discussion

The overall results of this study demonstrate the added value of 3D T2 imaging in evaluation of ECE. Our suggested quantitative parameters of 3D contact and 3D surface were both superior to previously reported tumour contact length measured in axial plane on 2D T2. 3D reads also increased sensitivity whilst maintaining high specificity by means of subjective scoring, but this did not reach significance. However, 3D reads significantly increased the mean Likert score in cases positive for ECE and showed a trend towards lower mean score in negative cases, reflecting increased reader confidence, which may also be important from a reporting standpoint. Finally, the optimal threshold for capsular contact predicting ECE differed between low- and high-grade tumours, which has not been reported previously and is potential of key clinical importance.

Our results for capsular contact are in agreement with the results of previous work which showed a strong association between the MRI-determined contact length and ECE. However, previous studies have measured contact only in the axial plane on conventional T2 axial images [16–18]. Our results showed that contact length measured with 3D T2 significantly improved sensitivity from 59.6 to 73.7% whilst maintaining a high specificity at 87.8%. This confirmed our hypothesis that 3D would allow us to measure the true maximal length of capsular contact, as this is not necessarily the longest in the axial plane. The sensitivity for 3D-measured surface area contact was even higher at 82.5% (2D = 59.6%), but with a lower specificity of 71.4% (2D = 87.8%). 3D contact and 3D surface showed excellent reliability (ICC > 0.95) and substantial to almost perfect agreement (*k* = 0.79 and 0.92, respectively) which is concordant with the previous work reporting substantial agreement for linear contact measurements on axial T2 imaging (*k* = 0.70) [18]. 3D reformats take a few seconds to load and finding the longest contact/line generally takes under a minute. 3D parameters may only be needed as a problem solver in borderline cases, meaning the overall impact on workflow would be negligible, especially in the light of significantly increased reader confidence. Although 3D-measured surface requires some additional time due to calculations, it may prove particularly useful when high-sensitivity readings are required.

Regarding contact length thresholds, our numbers of 10.5 mm for 3D contact and 13.5 mm for axial 2D contact are between the axial 2D thresholds previously reported by Rosenkrantz et al (6 mm) and Baco et al (20 mm) [17, 18] but similar to the results of Woo et al (14 mm) [16]. The reasons may relate to study design; for instance, the Baco et al [17] study was performed at 1.5 T and Rosenkrantz et al [18] made radio-pathological correlations on a side-specific basis whereas we made these on a lesion-specific basis. Moreover, the latter created a single straight line between the two outer margins of the lesion’s capsular contact rather than total (curved) contact, which likely underestimated contact and may explain their lower threshold. Interestingly, we noticed a significant difference between contact lengths and thresholds in the axial and the vertical planes (coronal or sagittal) at 13.5 mm versus 5.5 mm, which suggests that tumours tend to spread along the capsule more in the transverse than vertical plane, in accordance with earlier work by McNeal et al [34]. Case mix may further explain the variations in literature-reported capsular contact required for ECE.

Our finding that the optimal capsular contact threshold differs between high-grade (grade group 3–5) and low-grade (grade group 1–2) tumours is clinically important. HGG tumours overall showed significantly more ECE than LGG (88% vs 44%). Although HGG tumours had a longer mean capsular contact than LGG (15.3 mm vs 9.0 mm), the finding appears to be independent of tumour size, with a marked difference also observed when corrected for a contact length of ≤ 10 mm (41.6% biopsy-confirmed HGG index lesions showing established ECE vs. 3% LGG). Consequently, in HGG tumours, a PPV of 90.9% was reached at ≥ 5 mm, whereas a PPV of 90.4% was only achieved at a cutoff at ≥ 12.5 mm in LGG tumours. Although not previously reported in MRI studies, the finding that aggressive cancers require less capsular contact for extracapsular spread is perhaps expected: Partin nomograms incorporate biopsy Gleason score as a predictor for ECE at radical prostatectomy [35]. Similar conclusions can also be deduced from the recent study of Matsuoka et al where 73.3–74.1% of patients with a biopsy Gleason score of ≤ 7 had over-staged tumours in comparison with 35.7–44.4% of those with a biopsy Gleason score of ≥ 8 when a PI-RADS 2.0 cutoff at 10 mm was used as staging criteria [19]. This is an important consideration in the context of performing MRI pre-biopsy, when pathological information is yet to be obtained; thus, it may be appropriate to re-evaluate for the likelihood of ECE when biopsy results become available.

**Fig. 4:**
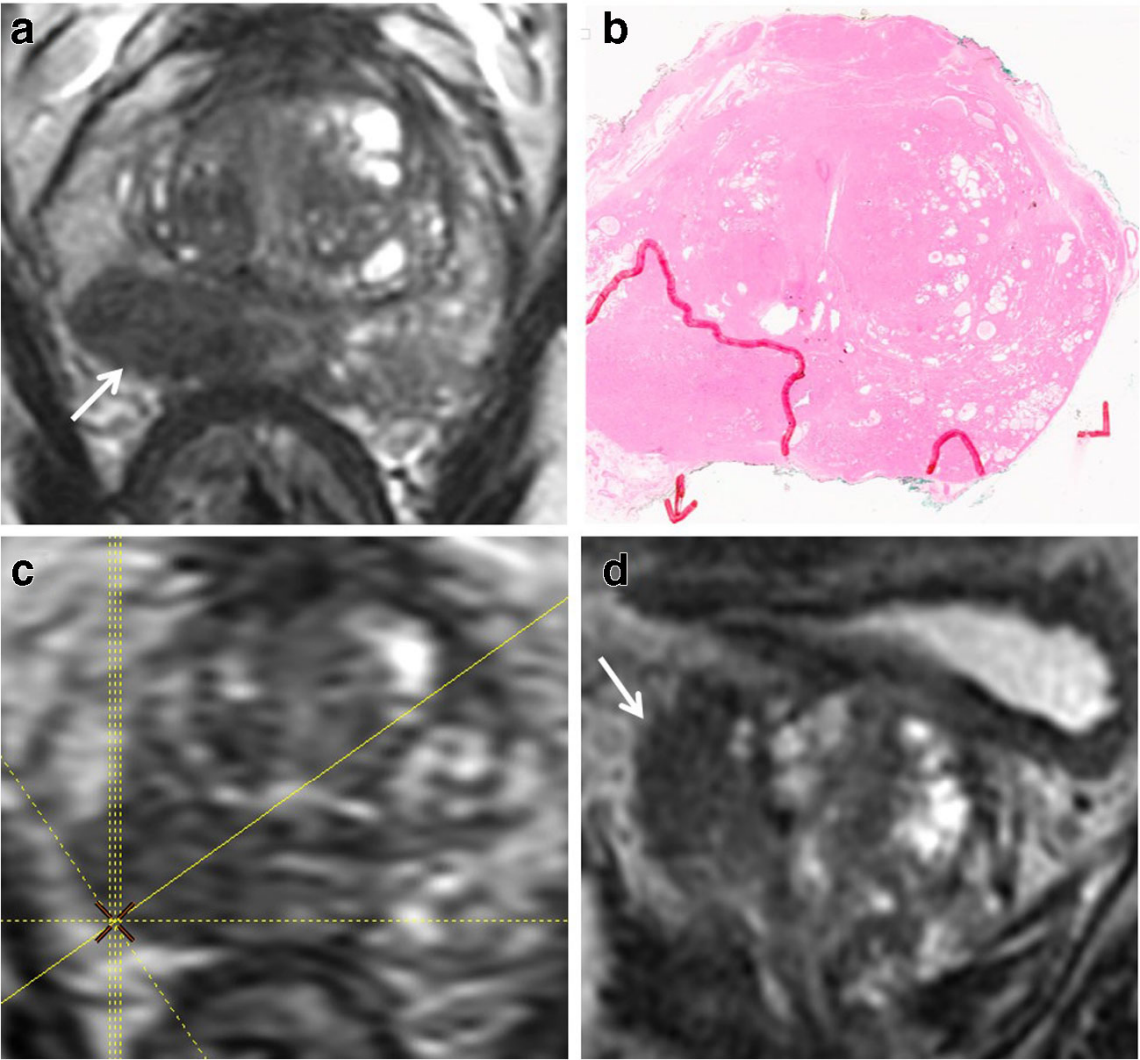
Cube imaging highlights macroscopic ECE. 65-year-old, PSA 12.4 ng/ml. **a**, **b** Axial T2WI shows right base PZ lesion with broad capsular contact (arrow in **a**). Histopathology slide from prostatectomy confirms Gleason 3 + 4 tumour at the right base PZ, with established extra-prostatic extension, pT3a disease (arrow). **c**, **d** Axial oblique reformat (**d**) to the lesion (plane in **c**) shows irregularity consistent with macroscopic ECE (arrow)

**Fig. 5:**
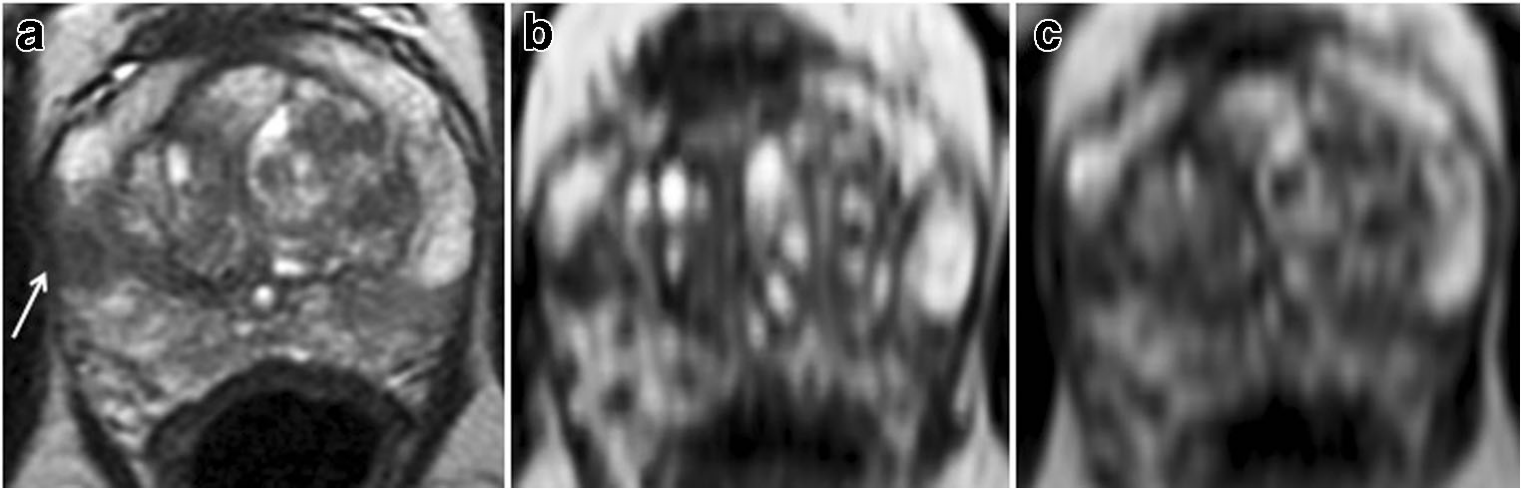
Cube imaging suggests less capsular contact. 65-year-old, PSA 5.85 ng/ml. **a** Axial T2WI shows mid gland lateral PZ right lesion (arrow) with apparent broad capsular contact of 12 mm. **b**, **c** Axial thin-sliced cube reformat at the equivalent 2 consecutive levels suggests a more minimal capsular contact of 5 mm. Prostatectomy showed a dominant mid gland PZ Gleason 4 + 4 = 8 lesion, measuring 8 × 6 mm but organ-confined (pT2)

**Fig. 6:**
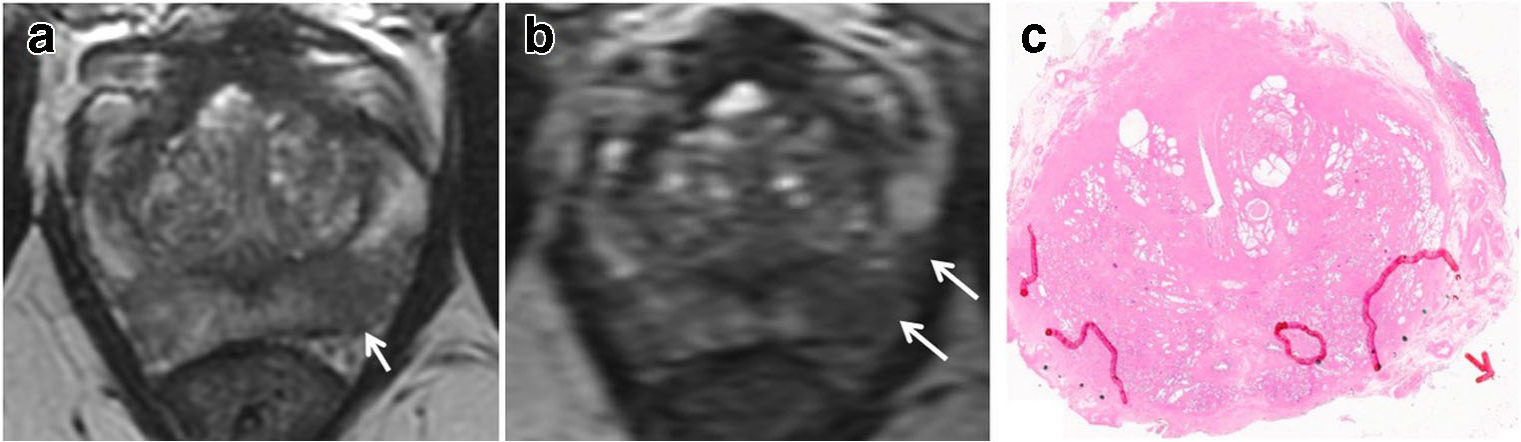
Cube imaging suggests increased capsular contact. 68-year-old, PSA 15.36 ng/ml. **a** Axial T2WI shows mid gland left PZ lesion (arrow) with minimal capsular contact posteriorly and no capsular contact laterally (arrow). **b** Axial thin-sliced cube reformat suggests far more extensive capsular contact extending laterally and anteriorly (arrows). **c** Histopathology slide from prostatectomy shows multifocal tumour with the dominant focus in the left mid gland, Gleason score 4 + 5 = 9, with established ECE, pT3a (arrow)

In terms of subjective scoring, two previous studies have compared 2D T2W to 3D T2W for predicting ECE. The results of the study by Itatani et al, who reconstructed using multiple planes at 1.5 T (slice thickness/gap 4/0 mm), are in concordance with ours [26]. Conversely, Rosenkrantz et al reported only a non-significant trend to increased sensitivity with the 3D method [25]. However, reformats were only performed in the three standard planes and using a slice thickness of 3 mm to match 2D T2, possibly underutilising the benefits of 3D imaging. We used the advantage of 3D imaging and reconstructed a plane perpendicular to the tumour-capsule interface similarly as in evaluating rectal or cervical cancer, where an oblique axial plane is recommended in order to avoid partial volume–related staging inaccuracy [23, 24] (Fig. 4). Additionally, the axial plane cube reformats afforded thinner axial slice (1 mm vs 3 mm) which resulted in less partial voluming and for more accurate delineation of capsular contact (Figs. 5 and 6).

Our study has a number of limitations, including its single-centre and retrospective design. Our cohort is limited by its relatively small numbers, particularly for the sub-analysis of HGG versus LGG at a capsular contact of ≤ 10 mm. Additionally, the reader may have been biased by knowledge of the existing literature on capsular contact [16–19], which may explain the limited additional benefit of measuring capsular contact over subjective scoring alone, with the latter being biased by the former. However, of note, previous work by Outwater et al [36] and Yu et al [37] did not show improvement when adding contact length to morphological criteria. In addition, readers in the earlier reported studies [16–18] are also likely to have been aware of the PI-RADS version 1 guidelines, which suggested broad capsular contact (> 15 mm) as a criterion for lesion scoring [38]. We did not compare signal-to-noise ratio (SNR) and overall image quality between the 2D and 3D imaging as this was not the principal aim of our work. However, we did demonstrate added value of 3D imaging in evaluating ECE, which together with other studies [26–28, 39] form a basis for consideration of using abbreviated MRI prostate protocols. Moreover, there is emerging data that a single acquisition of 3D T2W rather than time-consuming conventional 2D T2 in three dedicated planes may produce results of equal diagnostic quality [25, 40].

In conclusion, our study showed significantly increased sensitivity and confidence in calling ECE with 3D T2 over conventional 2D T2. The capsular contact length threshold differed between low- and high-grade cancers, suggesting that MRI restaging should be considered in light of the histological information when MRI is performed pre-biopsy.

## Electronic supplementary material

ESM 1 (DOCX 467 kb)

## Abbreviations

DRE: Digital rectal examination
DWI: Diffusion-weighted imaging
ECE: Extracapsular extension
ERC: Endorectal coils
FSE: Fast spin echo
HGG: High-grade group
LGG: Low-grade group
MRI: Magnetic resonance imaging
PCa: Prostate cancer
PI-RADS: Prostate Imaging Reporting and Data System
T2W: T2-weighted
T2WI: T2-weighted imaging
