# Supplementary material for: Defining the incremental value of 3D T2-weighted imaging in the assessment of prostate cancer extracapsular extension
Source: Eur Radiol. 2019 Mar 18;29(10):5488–97. doi: 10.1007/s00330-019-06070-6 (PMC6719333; doi:10.1007/s00330-019-06070-6)
Supplement: Supplementary file 1 — (DOCX 467 kb) [file 330_2019_6070_MOESM1_ESM.docx]

**Supplemental Figure 1**

3D-contact is defined as maximum as maximal length of tumour in contact with prostatic capsule.

Distance **a**: 3D-contact

Distance **b**: 2^nd^ measurement used for 3D-surface calculation

Dashed line (**c**) indicating axial plane where 2D-contact is measured.

**Supplemental Tables**

**Supplemental Table 1:** Low grade subgroup. Performance of maximal tumour contact length determined on 3D T2WI (3D contact) at different thresholds.

| threshold | specificity | sensitivity | ppv | npv |
| --- | --- | --- | --- | --- |
| -Inf | 0.00 | 1.00 | 0.44 | NaN |
| 0.5 | 0.23 | 1.00 | 0.50 | 1.00 |
| 1.5 | 0.23 | 0.97 | 0.49 | 0.91 |
| 2.5 | 0.34 | 0.97 | 0.53 | 0.94 |
| 3.5 | 0.39 | 0.88 | 0.53 | 0.81 |
| 4.5 | 0.52 | 0.85 | 0.58 | 0.82 |
| 5.5 | 0.73 | 0.82 | 0.70 | 0.84 |
| 6.5 | 0.73 | 0.79 | 0.69 | 0.82 |
| 7.5 | 0.77 | 0.79 | 0.73 | 0.83 |
| 8.5 | 0.80 | 0.76 | 0.74 | 0.81 |
| 9.5 | 0.82 | 0.74 | 0.76 | 0.80 |
| 10.5 | 0.86 | 0.71 | 0.80 | 0.79 |
| 11.5 | 0.89 | 0.62 | 0.81 | 0.75 |
| 12.5 | 0.95 | 0.56 | 0.90 | 0.74 |
| 13.5 | 0.95 | 0.47 | 0.89 | 0.70 |
| 14.5 | 0.95 | 0.35 | 0.86 | 0.66 |
| 15.5 | 0.95 | 0.32 | 0.85 | 0.65 |
| 16.5 | 0.98 | 0.29 | 0.91 | 0.64 |
| 17.5 | 0.98 | 0.26 | 0.90 | 0.63 |
| 20.0 | 0.98 | 0.24 | 0.89 | 0.62 |
| 23.0 | 1.00 | 0.18 | 1.00 | 0.61 |
| 24.5 | 1.00 | 0.15 | 1.00 | 0.60 |
| 25.5 | 1.00 | 0.12 | 1.00 | 0.59 |
| 27.0 | 1.00 | 0.09 | 1.00 | 0.59 |
| 30.0 | 1.00 | 0.06 | 1.00 | 0.58 |
| 32.5 | 1.00 | 0.03 | 1.00 | 0.57 |
| Inf | 1.00 | 0.00 | NaN | 0.56 |

**Supplemental Table 2:** High grade subgroup. Performance of maximal tumour contact length determined on 3D T2WI (3D contact) at different thresholds.

| threshold | specificity | sensitivity | ppv | npv |
| --- | --- | --- | --- | --- |
| -Inf | 0.00 | 1.00 | 0.82 | NaN |
| 1.5 | 0.20 | 0.91 | 0.84 | 0.33 |
| 3.5 | 0.40 | 0.91 | 0.88 | 0.50 |
| 5.5 | 0.60 | 0.91 | 0.91 | 0.60 |
| 8.0 | 0.80 | 0.87 | 0.95 | 0.57 |
| 9.5 | 1.00 | 0.87 | 1.00 | 0.63 |
| 11.5 | 1.00 | 0.83 | 1.00 | 0.56 |
| 13.5 | 1.00 | 0.78 | 1.00 | 0.50 |
| 14.5 | 1.00 | 0.74 | 1.00 | 0.45 |
| 16.0 | 1.00 | 0.70 | 1.00 | 0.42 |
| 18.0 | 1.00 | 0.65 | 1.00 | 0.38 |
| 19.5 | 1.00 | 0.61 | 1.00 | 0.36 |
| 21.5 | 1.00 | 0.43 | 1.00 | 0.28 |
| 24.0 | 1.00 | 0.35 | 1.00 | 0.25 |
| 25.5 | 1.00 | 0.30 | 1.00 | 0.24 |
| 27.5 | 1.00 | 0.26 | 1.00 | 0.23 |
| 30.0 | 1.00 | 0.22 | 1.00 | 0.22 |
| 32.0 | 1.00 | 0.17 | 1.00 | 0.21 |
| 34.0 | 1.00 | 0.13 | 1.00 | 0.20 |
| 42.0 | 1.00 | 0.09 | 1.00 | 0.19 |
| 51.5 | 1.00 | 0.04 | 1.00 | 0.19 |
| Inf | 1.00 | 0.00 | NaN | 0.18 |
